# A Hierarchically Structured Composite Integrating a Biomass-Derived Magnetic Carbon Framework with Various Magnetic Phases, Exhibiting Outstanding Electromagnetic Wave Absorption Performance

**DOI:** 10.3390/molecules31101775

**Published:** 2026-05-21

**Authors:** Yutao Zhang, Jiawei Bi, Tiancheng Yuan, Shenpeng Xia, Minzhen Bao

**Affiliations:** 1 Zhejiang Key Laboratory of Soil Remediation and Quality Improvement, School of Environment and Resources, Zhejiang Agriculture and Forestry University, Hangzhou 311300, China; hsjavab@163.com; 2 Bamboo Home Engineering Technology Research Center of National Forestry and Grassland Administration, China National Bamboo Research Center, Hangzhou 310012, China; ytc_njfu@163.com (T.Y.); xsp@zafu.edu.cn (S.X.); 3 Sino-Spain Joint Laboratory for Agricultural Environment Emerging Contaminants of Zhejiang Province, School of Environment and Resources, Zhejiang Agriculture and Forestry University, Hangzhou 311300, China

**Keywords:** bamboo-derived porous carbon, sphere-like Fe_3_O_4_ nanostructures, effective absorption bandwidth, reflect loss, radar cross-section

## Abstract

A lightweight and high-efficiency microwave-absorbing material was developed via an in situ solvothermal pyrolysis strategy by anchoring sphere-like Fe_3_O_4_ nanostructures onto bamboo-derived porous carbon (BPC). The resulting composites preserve the intrinsic anisotropic honeycomb architecture of bamboo while introducing uniformly distributed magnetic nanoparticles, enabling synergistic dielectric–magnetic loss. Electromagnetic parameters, alongside impedance matching, were successfully modulated through the optimization of precursor concentrations. Of the evaluated materials, BPC-0.9 stood out for its intense attenuation, recording an RL_min_ of −45.17 dB at a 1.8 mm thickness. Furthermore, a significant effective absorption bandwidth of 6.65 GHz was attained by the BPC-0.6 sample at only 2.2 mm. Several factors contribute to the boosted efficiency, starting with conductive and interfacial polarization losses paired with multiple scattering events. Furthermore, magnetic loss components, encompassing eddy current effects as well as natural and exchange resonances, play a pivotal role in optimizing the material’s response. Furthermore, radar cross-section (RCS) modeling reveals a substantial reduction of 19.9 dB·m^2^, verifying the material’s viability for real-world stealth technologies. Our findings offer a straightforward methodology for fabricating magnetic carbon structures from biomass with adjustable dielectric responses, underscoring their potential in high-performance energy conversion and low-density microwave absorption.

## 1. Introduction

In recent years, electromagnetic technologies have advanced rapidly, demonstrating significant growth and broad potential for applications in electronic communication systems and military-related fields. At the same time, various issues associated with electromagnetic interference and radiation have drawn increasing public attention, including potential impacts on the immune and nervous systems, disturbances in the operation of precision instruments, and adverse effects on the growth and development of plants and animals. Therefore, engineering high-performance microwave attenuators (MAMs) has become a critical priority [1,2]. These materials are essential for safeguarding both biological entities and precision instrumentation against the detrimental interference associated with electromagnetic radiation [3,4]. In particular, there is an urgent demand for MAMs that combine low density, reduced matching thickness, strong attenuation capability, broad effective absorption bandwidth, excellent chemical stability, low cost, and a straightforward synthesis route [5,6]. During the past decade, numerous types of MAMs have been widely explored, including metal powders, magnetic ferrites, carbon-based materials metal oxides and carbides [7,8].

Among these candidates, carbon-based materials have long been regarded as highly promising lightweight MAMs due to their exceptionally low density and excellent electrical stability [9,10]. At the early stage of research development, biomass-derived carbon materials, as an emerging member of the carbon material family, attracted considerable attention because of their unique porous structures and abundant surface area, in addition to the common advantages shared by other carbonaceous materials [11,12]. In general, effective impedance matching together with multiple energy dissipation mechanisms constitutes the fundamental basis for achieving excellent MWA performance in absorbing materials [13,14]. However, biomass materials that rely solely on dielectric loss mechanisms are typically insufficient to achieve both strong attenuation and wide absorption bandwidth [15,16]. The introduction of strong magnetic loss pathways, including exchange resonance and eddy current phenomena, has made the hybridization of carbon absorbers with magnetic components a focal point of current study [17,18]. Such configurations effectively augment the overall absorption profile by balancing dielectric and magnetic contributions [19,20,21,22].

As a representative ferrite material, Fe_3_O_4_ exhibits strong magnetocrystalline anisotropy and high saturation magnetization, together with remarkable mechanical hardness, excellent oxidation resistance, and superior corrosion resistance [23,24]. Furthermore, Fe_3_O_4_ exhibits a relatively high Snoek’s limit, large complex permeability, and strong magnetic loss in the high-frequency region. These characteristics make it suitable for integration with carbon-based matrices through various modification strategies, enabling the construction of composites with enhanced microwave absorption performance, particularly in the high-frequency range while maintaining a relatively thin matching thickness [25,26,27]. Nevertheless, most of the previously reported approaches require stringent reaction conditions and involve complicated, time-consuming procedures [28,29,30]. Moreover, the resulting materials often struggle to simultaneously achieve both strong attenuation and wide absorption bandwidth, which significantly limits the practical deployment of microwave-absorbing materials.

In this work, a novel sphere-like Fe_3_O_4_ magnetic structure composed of numerous nanoscale particles and a mesoporous architecture was successfully incorporated into bamboo carbon through a one-step in situ solvothermal approach. Relative to pristine bamboo derived carbon, the synthesized Fe_3_O_4_/bamboo carbon hybrid nanocomposites demonstrate markedly improved microwave absorption capability across the frequency range. In addition, the RL values and EAB of the nanocomposites can be readily tuned by regulating the loading amount of Fe_3_O_4_ magnetic nanospheres and the filler proportion in the microwave-absorbing system. Furthermore, a comprehensive MWA mechanism combining effective impedance matching with multiple loss processes has been proposed to clarify the propagation behavior of incident electromagnetic waves and their subsequent energy dissipation within the lightweight absorber.

## 2. Results and Discussion

**Figure 1:**
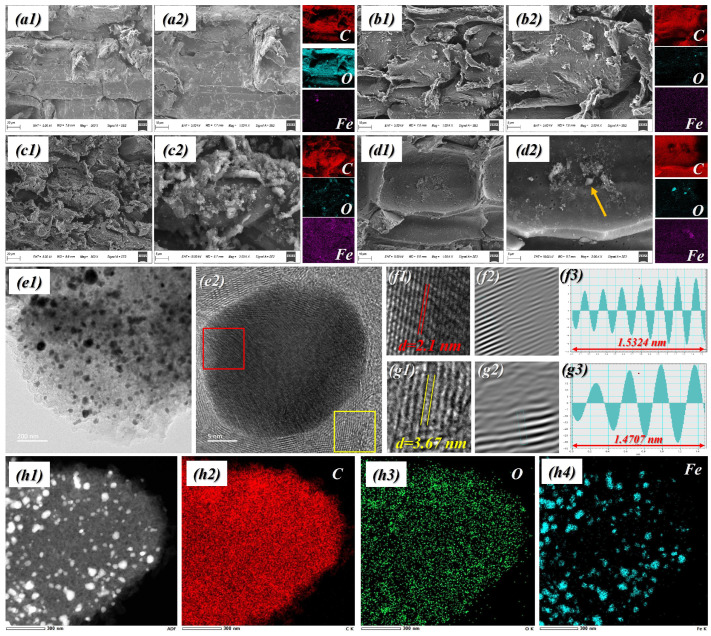
Microscopy results for the obtained samples: (**a1**,**a2**) Micromorphology of BPC; (**b1**,**b2**) Micromorphology of BPC-0.3; (**c1**,**c2**) Micromorphology of BPC-0.6; (**d1**,**d2**) Micromorphology of BPC-0.9; (**e1**,**e2**,**f1**–**f3**,**g1**–**g3**,**h1**–**h4**) TEM images of BPC-0.9.

This porous Fe_3_O_4_/BPC material with a honeycomb structure was prepared through in situ impregnation, vacuum drying and high-temperature pyrolysis [31]. The resulting BPC exhibited a low density of 0.362 g/cm^3^ and was light enough to rest on a leaf without difficulty (Figure S1). Oriented pore channels with widths near 60 μm formed a honeycomb-like architecture, enabling multiple routes for EMW reflection. As presented in Figure 1(a1,a2), SEM observations of carbon obtained from untreated bamboo reveal a highly ordered porous architecture that largely retains the intrinsic structure of the native material, characterized by elongated channels and well-preserved cell walls [32]. The surface is comparatively smooth and free of extraneous features, with no evidence of nanoparticle deposition or secondary phases, which is consistent with the absence of Fe_3_O_4_ incorporation and the lack of Fe signals in the corresponding EDS analysis. As illustrated in Figure 1(b1–d2), the BPCs display characteristic morphological features. For instance, in the BPC-0.9 sample (Figure 1(d1,d2)), numerous fine spherical particles are distributed within the lumens of parenchyma cells. Elemental mapping via EDS verifies that these particulates are associated with Fe signals, thereby prompting further TEM characterization to elucidate the crystalline structure of the magnetic nanoparticles. A large number of iron nanoparticles, around 50 nm in diameter, were homogeneously distributed across the carbon scaffold. Thus, it is evident that iron particles were firmly anchored to the BPC, which can markedly enhance interfacial quality and thereby promote polarization. The TEM characterization results are presented in Figure 1(e1,e2,f1–f3,g1–g3). As observed in Figure 1(e2), the metallic nanoparticles are enveloped by a combination of graphitized carbon layers and amorphous carbon. Detailed examination of the lattice fringes (Figure 1(f3,g3)) reveals that the interplanar spacing in Figure 1(f2) is approximately 2.1 nm, which can be assigned to the (400) plane of Fe_3_O_4_, whereas the spacing in Figure 1(g2) is about 3.67 nm, corresponding to the (200) plane of carbon. These findings provide clear evidence that the magnetic nanoparticles are effectively anchored onto the bamboo-derived carbon matrix. This interpretation is further supported by the elemental mapping results shown in Figure 1(h1–h4).

**Figure 2:**
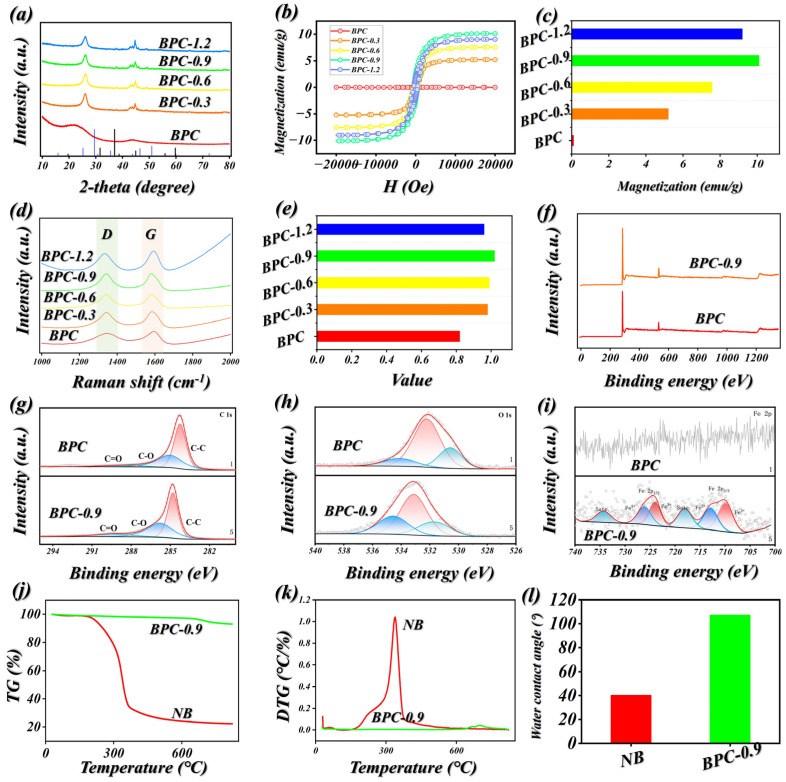
(**a**) XRD curves of BPCs specimens. (**b**,**c**) VSM curves of BPC specimens and the maximum saturation magnetization. (**d**,**e**) Raman spectrogram of BPC specimens. (**f**) XPS spectrogram of BPC and BPC-0.9. (**g**–**i**) C1s, O1s and Fe2p spectrogram of BPC and BPC-0.9. (**j**,**k**) TG and DTG curves of Natural Bamboo (NB) and BPC-0.9. (**l**) water contact angle of NB and BPC-0.9.

Figure 2a presented the XRD curves of BPCs, the XRD spectra of the prepared composites exhibit a series of distinct diffraction peaks that can be accurately matched with the standard reference pattern of Fe_3_O_4_, thereby verifying the formation of magnetite during the carbonization process. Distinct diffraction maxima appear at around 2θ = 30.1°, 35.5°, 43.1°, 53.4°, 57.0°, and 62.6°, which can be assigned to the (220), (311), (400), (422), (511), and (440) crystallographic planes of Fe_3_O_4_, respectively. By comparison, the unmodified carbonized sample (BPC) shows no discernible diffraction features associated with Fe_3_O_4_, whereas these reflections are prominently present in the BPC-X series, demonstrating the effective introduction of Fe_3_O_4_ into the carbon framework. Furthermore, the absence of additional diffraction peaks implies that the magnetic phase possesses a high degree of purity without detectable impurities. As presented in Figure 2b,c, the pristine bamboo-derived sample (BPC) exhibits an extremely low saturation magnetization of 0.01 emu/g, reflecting its essentially non-magnetic nature and negligible contribution to magnetic behavior. Following the in situ formation of iron (III) oxide (Fe_3_O_4_) nanoparticles on the bamboo surface, all modified specimens display a marked increase in saturation magnetization, demonstrating the successful incorporation of magnetic Fe_3_O_4_ within the bamboo framework. As the concentration of the iron salt precursor increases, the magnetic response of the composites initially strengthens and then declines. Specifically, when the precursor concentration rises from 0.3 mol/L to 0.9 mol/L, the saturation magnetization values of BPC-0.3, BPC-0.6, and BPC-0.9 increase to 5.21, 7.59, and 10.11 emu/g, respectively. This enhancement can be attributed to the elevated precursor concentration, which facilitates the nucleation and growth of Fe_3_O_4_ crystals, leading to a higher loading amount or improved crystallinity of magnetic particles and thus a stronger macroscopic magnetic response. However, a further increase in concentration to 1.2 mol/L results in a slight reduction in saturation magnetization to 9.02 emu/g for BPC-1.2. Such a decline is likely caused by excessive particle aggregation on the bamboo surface, which enlarges particle size and promotes a transition from single-domain to multi-domain structures, or by intensified surface spin disorder, both of which weaken the magnetization per unit mass [26,33]. These findings suggest that the magnetic performance of bamboo-based Fe_3_O_4_ composites can be effectively tuned by adjusting the precursor concentration, with 0.9 mol/L identified as the optimal condition in this study. As presented in Figure 2d,e, following carbonization at 1000 °C, the I_D_/I_G_ values of all specimens display a clear dependence on composition. The pristine bamboo-derived carbon (BPC) shows an I_D_/I_G_ ratio of 0.82, indicative of a typical disordered carbon framework. With increasing iron salt precursor concentration, the I_D_/I_G_ ratios of the composites rise initially and then decline, reaching 0.98, 0.99, and 1.02 for BPC-0.3, BPC-0.6, and BPC-0.9, respectively, before decreasing slightly to 0.96 for BPC-1.2. This behavior originates from the combined catalytic and defect-inducing roles of Fe_3_O_4_-derived iron-based nanoparticles during high-temperature treatment [34,35,36]. At 1000 °C, Fe_3_O_4_ anchored on the bamboo surface undergoes carbothermal reduction, generating metallic iron or Fe_3_C in situ. These species act as catalysts for the conversion of amorphous carbon into graphitized structures [37]. However, rather than forming highly ordered graphite, this process produces numerous defective configurations, including nanocrystalline graphitic domains, edge imperfections, and catalyst-carbon interfacial regions, which intensify the Raman D band. As the precursor concentration increases up to BPC-0.9, the growing number of catalytic sites promotes the formation of more defect-rich carbon structures, leading to a continuous increase in the I_D_/I_G_ ratio. In contrast, excessive iron loading in BPC-1.2 causes particle aggregation, diminishing catalytic effectiveness and potentially inducing localized over-graphitization, thereby reducing the relative defect density within the carbon matrix and resulting in a lower I_D_/I_G_ value [38,39,40,41,42,43,44].

XPS was employed to investigate the surface elemental composition and chemical states of the carbonized materials. As depicted in Figure 2f–i, the survey spectrum of BPC shows no detectable Fe 2p signal, verifying the absence of iron species in the carbon framework. In contrast, the high-resolution Fe 2p spectrum of BPC-0.9 presents two characteristic spin–orbit splitting peaks located at approximately 711.2 eV (Fe 2p_3/2_) and 724.6 eV (Fe 2p_1/2_), along with a satellite feature near 719.0 eV. These spectral signatures are typical of Fe^3+^ in an octahedral coordination environment, confirming that iron has been successfully incorporated into the carbon matrix, predominantly in the form of iron oxides. After iron incorporation, the BPC-0.9 sample exhibits a slight increase in the proportion of sp^2^ carbon alongside a pronounced reduction in oxygen-related signals, suggesting that iron species facilitate deoxygenation and promote the development of graphitic domains during high-temperature treatment. Further evidence is provided by the O1s spectra: BPC displays a single peak at ~532.5 eV attributed to C-O bonds, whereas BPC-0.9 reveals an additional component at ~530.2 eV corresponding to Fe-O bonds within the oxide lattice. This observation aligns well with the Fe 2p analysis, collectively demonstrating the formation of a well-defined iron oxide–carbon interface.

Additional evaluations of flame-retardant performance were performed for both natural bamboo and the BPC-0.9 sample. The corresponding TG and DTG profiles are presented in Figure 2j,k. At 822 °C, the untreated bamboo retains only 22% of its mass as residual carbon, whereas BPC-0.9 exhibits a significantly higher char yield of 92.55%. In addition, the DTG curve indicates that BPC-0.9 exhibits a lower maximum mass loss rate than that of pristine bamboo. The untreated bamboo surface exhibits a water contact angle of 38°, demonstrating clear hydrophilic behavior due to the high density of polar oxygen-containing functional groups present (Figure 2l). After carbonization at 1000 °C, the contact angle of the BPC-0.9 sample rises markedly to 107°, indicating a transition to hydrophobicity. This transformation can be attributed to two primary factors. On one hand, elevated temperatures induce the decomposition and removal of surface oxygen-containing groups, while simultaneously promoting graphitization of the carbon framework, leading to the formation of low-surface-energy, nonpolar sp^2^ carbon structures. On the other hand, the carbonization process generates a hierarchical micro–nano architecture composed of porous carbon and iron-based particles, which increases surface roughness [45,46]. According to the Cassie–Baxter model, this rough morphology traps air pockets at the interface, thereby enhancing the apparent contact angle.

**Figure 3:**
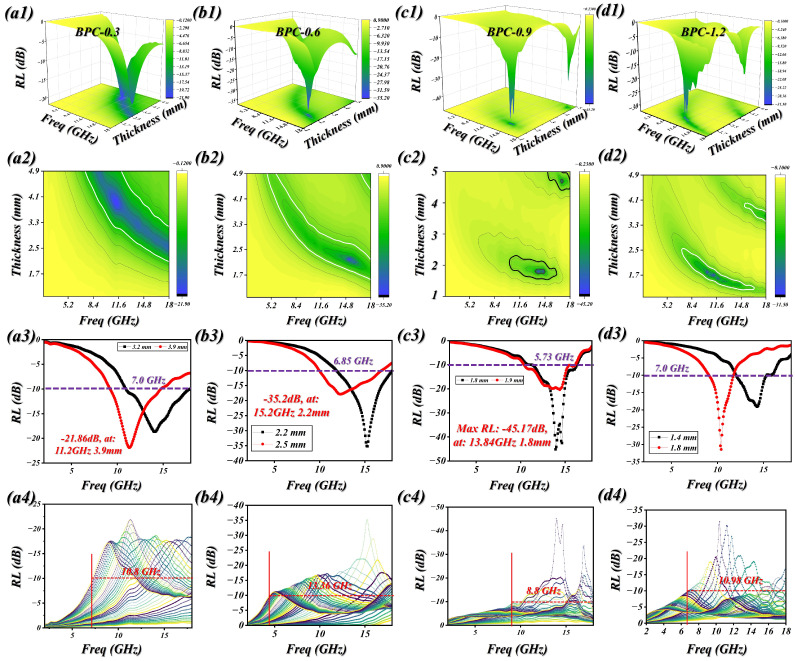
2D and 3D RL images of BPCs. (**a1**–**a4**) BPC-0.3, (**b1**–**b4**) BPC-0.6, (**c1**–**c4**) BPC-0.9, (**d1**–**d4**) BPC-1.2.

The electromagnetic wave dissipation capabilities of different samples are shown in Figure 3 and Figure S2. BPC exhibits poor microwave dissipation, recording a minimum reflection loss (RL_min_) of just −2.95 dB at 4.8 mm due to insufficient dielectric–magnetic interactions. Conversely, BPC-0.3 shows a significant performance boost, with an RL_min_ of −21.86 dB at 3.9 mm and an EAB_max_ reaching 7.0 GHz at 3.2 mm (Figure 3(a1–a3)). This enhancement is attributed to the successful integration of synergistic loss mechanisms and refined impedance matching within the composite [47,48]. As the precursor concentration is further elevated, the samples BPC-0.6 and BPC-0.9 are produced (Figure 3(b1,c1)). Among them, BPC-0.6 delivers optimal performance, achieving a RL_min_ of −35.2 dB at a thickness of 2.2 mm and an EAB_max_ as wide as 6.85 GHz. This marked improvement arises from the appropriate incorporation of magnetic nanoparticles, which enhances magnetic loss and interfacial polarization while also facilitating better impedance matching with free space. Meanwhile, the enriched heterointerfaces and the formation of a conductive network contribute to intensified multiple reflections and energy dissipation of electromagnetic waves, allowing high-efficiency absorption even at a relatively small thickness. In the case of BPC-0.9, a RL_min_ of −45.17 dB is achieved at a matching thickness of 1.8 mm, accompanied by a EAB_max_ of 5.73 GHz. The enhanced attenuation performance primarily stems from the higher incorporation of magnetic nanoparticles, which intensifies both magnetic loss and interfacial polarization. Nevertheless, excessive loading can induce a certain degree of impedance mismatch, leading to a slight reduction in effective bandwidth despite the improved attenuation capability. For BPC-1.2, the minimum reflection loss is −31.43 dB at a thickness of 1.8 mm, while the maximum effective absorption bandwidth reaches 3.34 GHz at a reduced thickness of 1.4 mm. The inferior performance relative to BPC-0.9 is mainly ascribed to the excessive incorporation of magnetic species, which disturbs the synergy between dielectric and magnetic dissipation and consequently worsens impedance matching. In addition, the tendency of nanoparticles to aggregate at higher loading levels diminishes the effective interfacial area and obstructs the development of a continuous conductive network, thereby suppressing interfacial polarization and multiple scattering processes. Consequently, despite the presence of considerable attenuation capability, the overall absorption efficiency and bandwidth are adversely affected. As illustrated in Figure 3(a4–d4), by tuning the matching thickness, the BPC samples are capable of covering nearly the entire Xand Ku-band frequency ranges, demonstrating their outstanding electromagnetic wave absorption performance across multiple frequency bands.

**Figure 4:**
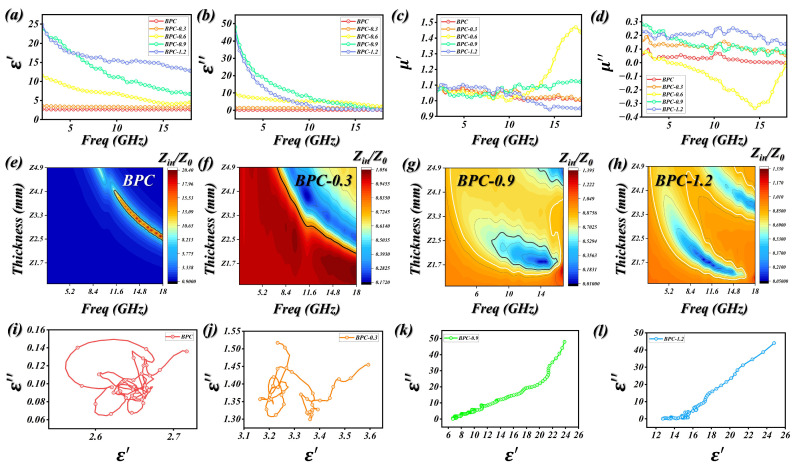
EM parameters of BPC specimens in the range of 2–18 GHz. (**a**) ε′, (**b**) ε″, (**c**) μ′, (**d**) μ″. (**e**–**h**) Z_in_/Z_0_ of BPCs. (**i**–**l**) Cole–Cole curves of BPCs.

Broadly speaking, a material’s capacity for EMW attenuation is intimately linked to its intrinsic electromagnetic characteristics [49,50]. These are represented by the relative complex permittivity (εr = ε′ − jε″) alongside the relative complex permeability (μr = μ′ − j μ″), which dictate the overall dielectric and magnetic responses. From the perspective of electromagnetic energy transformation, the real components ε′ and μ′ of an absorber describe its ability to store electric and magnetic energies, respectively. By contrast, the imaginary components ε″ and μ″ correspond to the ability of the material to dissipate electromagnetic energy, reflecting dielectric loss and magnetic loss, respectively. The variations in ε′, ε″, μ′, μ″, as well as the dielectric loss tangent (tan δ_ε_) and magnetic loss tangent (tan δ_μ_) for the BPC samples containing 30 wt% absorber as a function of frequency are presented in Figure 4a–d and Figure S3. Figure 4a illustrates the real component of the complex permittivity (ε′). In general, a suitable ε′ value is a crucial parameter for achieving effective EMW absorption in an ideal absorber. For the BPC-1.2 sample, the real component of the complex permittivity (ε′) exhibits a gradual decline as the frequency increases, decreasing from about 24.7 to nearly 12.8 over the frequency range of 2–18 GHz. An excessively large ε′ value can negatively influence impedance matching behavior. In contrast, the BPC-0.6 sample calcined at 1000 °C and the BPC-0.9 sample with surface polymer modification both exhibit moderate ε′ values, which facilitates the penetration of electromagnetic waves into the absorber and thereby improves absorption conditions. Generally, the dielectric loss capacity of a medium is represented by the imaginary permittivity (ε″). This component originates from the integrated effects of charge carrier conduction and various polarization relaxations, both of which are essential for robust electromagnetic wave attenuation [14,51]. According to the free-electron theory, when conductive loss predominates in determining ε″, the imaginary permittivity typically declines as the frequency increases within the 2–18 GHz range. This frequency-dependent decrease is clearly observed for the BPC-0.9 and BPC-1.2 samples. In contrast, this behavior is not observed for the BPC and BPC-0.3 samples. When polarization relaxation takes place within the absorber, it usually gives rise to a distinct loss peak in the ε″ spectrum.

In general, electromagnetic wave-absorbing materials containing magnetic components such as Fe_3_O_4_ exhibit three primary magnetic loss mechanisms, including natural resonance, exchange resonance, and eddy current loss. Among these mechanisms, natural resonance generally occurs within the lower frequency region of 2–8 GHz. The corresponding complex permeability parameters of the samples are presented in Figure 4c,d. The real part of permeability (μ′) shows a sharp decline at lower frequencies, followed by a gradual decrease in the higher-frequency region, representing a characteristic dispersion phenomenon. As illustrated in Figure 4d, several resonance peaks appear in the μ″ curve of BPCs within the frequency range of 2–7 GHz, which can be assigned to the occurrence of natural resonance. Moreover, further resonance features are observed in BPC-0.9 between 11.9 and 18 GHz, corresponding to exchange resonance effects. It is noteworthy that the magnetic loss tangent (tan δμ = μ″/μ′) exhibits a variation trend that is fully consistent with that observed for μ″.

In general, effective microwave absorption requires the coordinated tuning of complex permittivity (ε_r_) and permeability (μ_r_) through multiple polarization pathways. The dielectric response is typically interpreted using Debye relaxation theory [52,53], where the real (ε′) and imaginary (ε″) components of permittivity are defined as follows (Formula (1)–(4)):

(1) $$\varepsilon_{r}=\varepsilon^{\prime }-{j\varepsilon }^{\prime \prime }=\varepsilon_{\infty }+\frac{\varepsilon_{s}-\varepsilon_{\infty }}{1+j2\pi f\tau }$$

(2) $$\varepsilon^{\prime }=\varepsilon_{\infty }+\frac{\varepsilon_{s}-\varepsilon_{\infty }}{1+{\left(2\pi f\right)}^{2}\tau^{2}}$$

(3) $$\varepsilon^{\prime \prime }=\frac{2\pi f\tau \left(\varepsilon_{s}-\varepsilon_{\infty }\right)}{1+{\left(2\pi f\right)}^{2}\tau^{2}}$$

(4) $${\left(\varepsilon^{\prime }-\frac{\varepsilon_{s}+\varepsilon_{\infty }}{2}\right)}^{2}+{\left(\varepsilon^{\prime \prime }\right)}^{2}={\left(\frac{\varepsilon_{s}-\varepsilon_{\infty }}{2}\right)}^{2}$$

The polarization characteristics can be inferred from the number of Cole–Cole semicircles constructed from ε′ and ε″. Each individual semicircle represents a distinct Debye-type relaxation process [54,55]. As illustrated in Figure 4i–l and Figure S6, the ε′ − ε″ plot of BPC does not exhibit a well-defined Cole–Cole semicircle; instead, it shows a nearly linear tail region. This feature suggests that the dielectric loss in BPC is dominated solely by conductive loss rather than polarization relaxation processes. The ε′ − ε″ plots reveal that BPC-0.3 exhibits two semicircular arcs, while BPC-0.6 presents three, and both BPC-0.9 and BPC-1.2 display four distinct semicircles. In all cases, a linear tail is also observed, indicating the contribution of conductive loss. Analogous to dielectric Cole–Cole behavior, each semicircle in the μ′ − μ″ plot corresponds to an individual magnetic resonance process [56,57]. For the BPC-0.3 sample, only two semicircles are observed, which can be attributed to the intrinsic resonance of BPC. In the cases of BPC-0.9 and BPC-1.2, two distinct semicircles are also present, further evidencing the coexistence of dual magnetic resonances originating from both the carbon matrix and Fe_3_O_4_. The quantitative description formulas for conductance loss and magnetic loss are shown in Equation (5), and the results are presented in Figure S5, indicating that conductive loss plays a dominant role.

(5) $$\varepsilon^{\prime \prime }\left(\omega \right)=\varepsilon_{P}^{\prime \prime }+\varepsilon_{c}^{\prime \prime }=\left(\varepsilon_{s}-\varepsilon_{\infty }\right)\frac{\omega \tau }{1+\omega^{2}\tau^{2}}+\frac{\sigma }{\varepsilon_{0}\omega }$$

Moreover, it is generally accepted that when the parameter (μ″(μ′)^−2^f^−1^ = 2πμ_0_σd^2/3^) remains nearly constant across the frequency range, the magnetic loss in the absorber can be primarily attributed to the eddy current effect (Figure S4). Each curve can be separated into a steeply decreasing region followed by a more gradual variation, suggesting that eddy current loss plays a crucial role in the overall magnetic dissipation. In general, exchange resonance is associated with nanoparticles smaller than 10 nm and typically emerges at frequencies above 10 GHz. Given that the Fe_3_O_4_ nanoparticles embedded in the matrix are on the order of ~10 nm and that μ″ shows a noticeable increase in the 15–18 GHz range, the contribution of exchange resonance should be taken into account. Accordingly, the overall magnetic loss originates from the combined effects of natural resonance, eddy current loss, and exchange resonance. The formulas for attenuation constant, impedance matching and wavelength theory are as shown in Equations (6)–(8).

*α* = (2^1/2^πf/*c*){μ″ε″ − μ′ε′ + [(μ″ε″ − μ′ε′)^2^ + (μ′ε″ + μ″ε′)^2^]^1/2^}^1/2^ = |*Im*(2πf/*c* ∗ (μ ∗ ε)^1/2^)| (6)

(7) $$\Gamma =\frac{Z_{\mathrm{in}}-Z_{0}}{Z_{\mathrm{in}}+Z_{0}}$$

(8) $$d=\frac{n\lambda }{4}=\frac{\mathrm{nc}}{4f\sqrt{\mu_{r}\varepsilon_{r}}}\left(n=1,3,5,\ldots \right)$$

Based on transmission line theory, optimal microwave absorption is achieved when the impedance of the material (Z_in_) closely matches that of free space (Z_0_), meaning the ratio |Z_in_/Z_0_| approaches unity. Under such impedance-matching conditions, the reflection coefficient at the material–air interface becomes minimal, allowing incident electromagnetic waves to effectively penetrate into the interior of the material. Figure 4e–h presents the variation of |Z_in_/Z_0_| for the BPCs, evaluated under the corresponding tR and tE conditions. It is evident that, for the BPC-0.9 sample, the vertical lines corresponding to the RL peak positions intersect the |Z_in_/Z_0_| curves at points that are closest to unity. This indicates that impedance matching has been effectively optimized through the tailored regulation of electromagnetic parameters and the rational design of wave propagation pathways. As electromagnetic waves penetrate and travel through a material, their energy is dissipated through both macroscopic destructive interference and inherent electromagnetic losses. When the absorber thickness corresponds to an odd multiple of one-quarter wavelength (λ/4), the reflected waves undergo phase cancelation, a phenomenon described by the quarter-wavelength (λ/4) model (as presented in Figure S7). The λ/4-f relationships are plotted to assess the role of destructive interference in the attenuation process. The theoretically calculated matching thickness shows a slight deviation from the experimental t_R_ and t_E_ values, which can be attributed to the irregular morphology of the powder samples. Furthermore, intrinsic electromagnetic loss arises from the high-frequency oscillation of dipoles induced by asymmetric microstructures under an external electromagnetic field, leading to the fundamental dissipation of electromagnetic energy.

**Figure 5:**
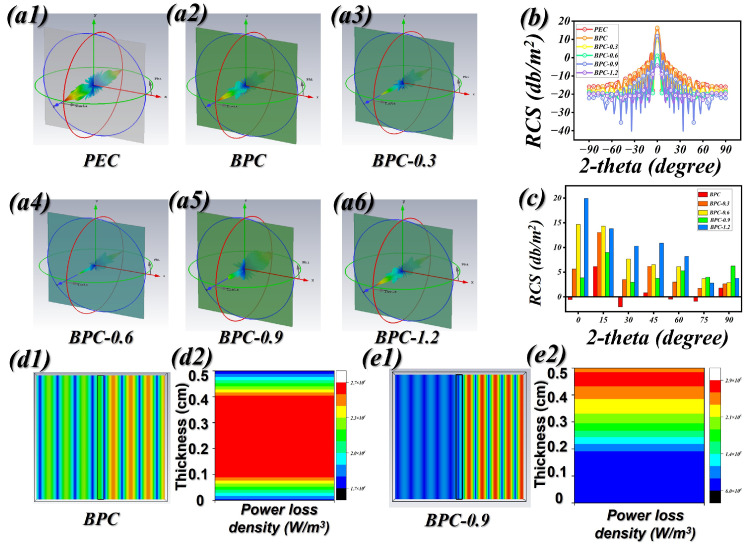
(**a1**–**a6**) RCS mode simulation results of BPCs. (**b**) The variation in the RCS values of the sample under different incident angles. (**c**) Bar chart showing the variation in RCS values at specific incidence angles. (**d1**,**e1**) Electric field intensity loss simulation diagram of BPC and BPC-0.9. (**d2**,**e2**) Energy loss density simulation diagram of BPC and BPC-0.9.

EMWA materials play a crucial role in decreasing the radar cross-section (RCS) of fighter aircraft, thereby enhancing stealth performance, increasing survivability under complex combat conditions, and lowering the probability of detection by hostile radar systems. In addition, CST Microwave Studio was employed to perform three-dimensional radar cross-section (RCS) analyses. These simulations encompassed both ideal perfectly conducting plates (PEC) and PECs coated with FSCA specimens, modeled within a realistic far-field scenario. Figure 5(a1) illustrates that the coating dimensions were defined as 90 mm × 90 mm × t mm, with a PEC of identical lateral size and 1.0 mm thickness positioned beneath it. The incident electromagnetic wave was simulated over a range of angles spanning 0° to 180°. Figure 5(a1–a6) presents the three-dimensional radar scattering pattern of the PEC, serving as the underlying substrate. Figure 5b depicts the radar cross-section (RCS) patterns as a function of the incident angle. Meanwhile, Figure 5c summarizes the RCS attenuation, calculated by subtracting the RCS of the bare PEC from that of the PEC coated with the BPC specimens. The BPC-1.2 specimen exhibits the most pronounced RCS attenuation, reaching 19.9 dB·m^2^. Figure 5c further illustrates that while the PEC coated with BPCs maintains the overall morphology of the reflected radar signals, the signal intensities vary. Notably, the BPC-0.9 and BPC-1.2 coatings generate the most intense scattering among the four samples, indicating their superior potential for electromagnetic wave absorption. Figure 5(d1,e1) present the simulated distributions of electric field intensity for BPC and BPC-0.9 at 6.88 GHz. In these models, the centrally positioned black rectangle denotes the coating layer. A periodic plane wave is introduced from the right side, propagating toward the absorber, and the resulting field variations are monitored on the opposite side. A comparison of the field responses reveals that the BPC sample exhibits the most pronounced variation on the incident side, indicating insufficient attenuation and thus the poorest electromagnetic absorption performance. In contrast, the BPC-0.9 sample shows a much weaker field disturbance, reflecting enhanced energy dissipation. Consequently, BPC-0.9 demonstrates the most effective capability for electromagnetic wave absorption. Energy loss density simulations were conducted to elucidate how electromagnetic waves are dissipated within the material. As illustrated in Figure 5(d2,e2), the spatial distribution of energy loss for both BPC and BPC-0.9 corresponds closely to the thickness ranges associated with their optimal reflection loss (RL) performance. This agreement indicates that the incident electromagnetic waves undergo progressive attenuation as they propagate into the absorber.

To begin with, the three-dimensional porous architecture facilitates repeated internal reflections of EMW. within the bamboo-derived carbon matrix. Meanwhile, charge transport within graphitic microcrystallites and electron hopping between adjacent domains generate significant conductive loss, which dominates the EMWA. process in the bamboo-based carbon framework. Furthermore, the incorporation of magnetic iron particles contributes to improved impedance matching. Meanwhile, their in situ formation ensures intimate contact with the bamboo-derived carbon matrix, strengthening the interfacial interaction and thereby intensifying interfacial polarization as well as the overall attenuation of EMW. Owing to the rational structural design and the straightforward in situ fabrication strategy, the material exhibits outstanding EMWA performance.

## 3. Materials and Methods

### 3.1. Materials

The bamboo strips are provided by Zhuqingjiaofa Co., Ltd. in Jiangling County, Lishui City, China. Iron (III) chloride hexahydrate (FeCl_3_·6H_2_O, ≥99%), ethylene glycol (EG, ≥99.5%), polyvinylpyrrolidone (PVP, Mw = 40,000), and sodium bicarbonate (NaHCO_3_, ≥99.7%) were purchased from commercial sources (Sigma-Aldrich, Shanghai, China) and used without further purification.

### 3.2. Fabrication of the Fe_3_O_4_/BPC Composites

For the preparation procedure, FeCl_3_·6H_2_O (2.0 mmol) together with PVP (0.5 g) was first introduced into a mixed solvent consisting of ethylene glycol (30 mL) and deionized water (5 mL). The suspension was subjected to ultrasonic treatment until a transparent and uniform solution was obtained, denoted as Solution A. In a separate step, NaHCO_3_ (5.0 mmol) was dispersed in ethylene glycol (10 mL) and fully dissolved under ultrasonication to generate Solution B. Subsequently, Solution B was slowly introduced into Solution A in a dropwise manner while the system was continuously agitated using vigorous magnetic stirring. After the addition was completed, the mixture was further stirred for 30 min to ensure complete homogenization. The resulting solution was then poured into a 50 mL Teflon-lined stainless-steel autoclave (Teflon, Chemours, Wilmington, DE, USA), tightly sealed, and subjected to solvothermal treatment in an electric oven (Thermo Fisher Scientific, Waltham, MA, USA) at 180 °C for 8 h. Following the reaction period, the autoclave was removed and permitted to cool to ambient temperature naturally. Centrifugation at 8000 rpm for 10 min was employed to isolate the dark precipitate from the reaction medium. To ensure the removal of unreacted precursors and contaminants, the obtained solid underwent several wash cycles using absolute ethanol and deionized water. Following this purification, the product was transferred to a vacuum oven (Thermo Fisher Scientific, Waltham, MA, USA) for 12 h of drying at 50 °C to yield the final material. Afterward, the synthesized Fe_3_O_4_ nanoparticles were combined with bamboo blocks in precursor solutions with concentrations of 0.3, 0.6, 0.9, and 1.2 mol/L. The mixtures were then subjected to high-temperature pyrolysis at 1000 °C for 2h, leading to the formation of Fe_3_O_4_/bamboo-derived porous carbon (BPC) composite materials.

### 3.3. Characterization

We systematically examined the morphology and microstructure of the prepared materials using scanning electron microscopy (SEM, Zeiss Sigma 500, Oberkochen, Germany) and transmission electron microscopy (TEM, JEOL JEM-2100, Tokyo, Japan). We further identified the crystalline phases of the samples using X-ray diffraction (XRD, Rigaku SmartLab, Tokyo, Japan) with Cu Kα radiation as the X-ray source. We analyzed the chemical states of the Fe_3_O_4_/biomass-derived carbon composites using X-ray photoelectron spectroscopy (XPS, Kratos AXIS Supra, Kyoto, Japan) and recorded Raman spectra with a LabRAM HR Evolution micro-Raman spectrometer. We evaluated the magnetic properties of the samples at 300 K using a vibrating sample magnetometer (VSM, Lakeshore 7404, Tokyo, Japan). To evaluate the MWA performance, we uniformly mixed the Fe_3_O_4_/biomass-derived carbon composites with paraffin wax at different filler loadings and then pressed the mixtures into standard toroidal samples with an inner diameter of 3.04 mm, an outer diameter of 7.00 mm, and a thickness of 3.04 mm. We measured the complex electromagnetic parameters of the samples at room temperature using a vector network analyzer (VNA, Keysight N5224A, Kyoto Japan) over the frequency range of 2–18 GHz. The RL can be calculated according to Formulas (9) and (10):

*Z_in_* = (*μ*/*ε*)^1/2^*tanh*[*j*(2*πfd*/*c*) ∗ (*μ* ∗ *ε*)^1/2^] (9)

*RL* = 20*log*_10_|(*Z_in_*− *Z*_0_)/(*Z_in_* + *Z*_0_)| (10)

where Z_in_ stands for the input impedance of the standard absorber, which is a crucial parameter in the analysis of absorption characteristics, Z_0_ represents the characteristic impedance of free space, a fundamental constant in the field of electromagnetics. The frequency of the electromagnetic wave, denoted by f, is a crucial factor in determining how the wave interacts with and behaves around the absorber. The thickness of the absorber, represented by d, significantly influences the efficiency and effectiveness of the absorption process. Additionally, c signifies the speed of the electromagnetic wave in free space.

### 3.4. RCS Simulation

The RCS simulations were carried out using CST Studio Suite. A 3D model of the target was first constructed according to the actual dimensions, retaining detailed features such as edges and corners. Material properties were assigned based on the real characteristics of the target: conductive or magnetic materials were given their respective conductivity and permeability, while composite materials were assigned effective permittivity and permeability derived from experimental data. To mimic a free-space environment, perfectly matched layers (PML) were applied around the model to prevent reflections. A plane wave excitation was used, with a frequency range corresponding to the radar operating band, and the incident angle and polarization were adjusted as required. The time-domain solver was selected to obtain wideband RCS data in a single simulation, and the mesh was refined in critical regions to ensure computational accuracy. After the simulation, the scattered field data were extracted from CST, and the RCS was calculated according to its definition at different observation angles. The RCS versus frequency curves were then plotted. The results showed that the scattering characteristics in the main directions agreed well with theoretical predictions, confirming the reliability of the model and simulation settings. This approach provides a straightforward way to evaluate the radar detectability of the structure under different angles and frequency bands, serving as a basis for subsequent structural optimization.

## 4. Conclusions

In summary, Fe_3_O_4_-decorated bamboo-derived porous carbon composites were successfully fabricated through a facile in situ solvothermal and high-temperature pyrolysis process. The incorporation of sphere-like Fe_3_O_4_ nanoparticles not only introduces magnetic loss but also enhances interfacial polarization and impedance matching, leading to significantly improved microwave absorption performance. Benefiting from the intrinsic anisotropic porous structure of bamboo and the well-dispersed magnetic nanophases, the composites exhibit strong attenuation capability and tunable absorption bandwidth across the X- and Ku-bands. Notably, the optimized BPC-0.9 achieves a remarkable RL_min_ of −45.17 dB at a thin thickness of 1.8 mm, while BPC-0.6 delivers a wide EAB of 6.65 GHz. The integration of diverse mechanisms—ranging from multiple scattering and conductive loss to natural and exchange resonance—drives the material’s superior response. Beyond absorption, the developed energy conversion prototype suggests a strategic path for secondary energy use, expanding the scope of multifunctional designs. By offering a sustainable and reproducible strategy for fabricating high-efficiency absorbers, this work accelerates the deployment of biomass-based composites in radar evasion and environmental electromagnetic safety.

## Data Availability

The original contributions presented in this study are included in the article/Supplementary Materials. Further inquiries can be directed to the corresponding authors.

## Supplementary Materials

The following supporting information can be downloaded at: https://www.mdpi.com/article/10.3390/molecules31101775/s1, Figure S1: Place the bamboo strips on the leaves; Figure S2: 2D and 3D RL results of BPC; Figure S3: (a) Dielectric loss tangent angle and (b) magnetic loss tangent angle of BPCs; Figure S4: AC and C0 results of BPCs; Figure S5: Conductive loss and polarization loss of different BPC specimens; Figure S6: Cole-Cole curves of BPC-0.6; Figure S7: One-quarter wavelength model of BPC-0.9; Table S1: Recent progress on biomass derived caron materials for microwave absorption, with a comparison to this work.
