# Supplementary material for: A Hierarchically Structured Composite Integrating a Biomass-Derived Magnetic Carbon Framework with Various Magnetic Phases, Exhibiting Outstanding Electromagnetic Wave Absorption Performance
Source: Molecules. 2026 May 21;31(10):1775. doi: 10.3390/molecules31101775 (PMC13209535; doi:10.3390/molecules31101775)
Supplement: Supplementary file 1 [file molecules-31-01775-s001.zip › molecules-4318438-supplementary.pdf]

# *A hierarchically structured composite integrating a biomass-derived magnetic carbon framework with various magnetic phases, exhibiting outstanding electromagnetic wave absorption performance*

***Yutao Zhang [1,3]*, Jiawei Bi [1,3] , Tiancheng Yuan [2], Shenpeng, Xia [2], Minzhen Bao [2,*]***

*1 Zhejiang Key Laboratory of Soil Remediation and Quality Improvement, School of Environment and Resources, Zhejiang Agriculture and Forestry University, Hangzhou 311300, P. R. China;* zhangyt2022@zafu.edu.cn *(Y.Z.) ;*hsjavab@163.com *(J.B.);*

*2 Bamboo Home Engineering Technology Research Center of National Forestry and Grassland Administration, China National Bamboo Research Center, Hangzhou 310012, China;* ytc_njfu@163.com *(T.Y.);* xsp@zafu.edu.cn *(X.X) ;* baominzhen@caf.ac.cn *(M.B.)*

*3 Sino-Spain Joint Laboratory for Agricultural Environment Emerging Contaminants of Zhejiang Province, School of Environment and Resources, Zhejiang Agriculture and Forestry University, Hangzhou 311300, P. R. China;*

* *corresponding author:*

zhangyt2022@zafu.edu.cn *(Y.Z.)*

baominzhen@caf.ac.cn *(M.B.)*

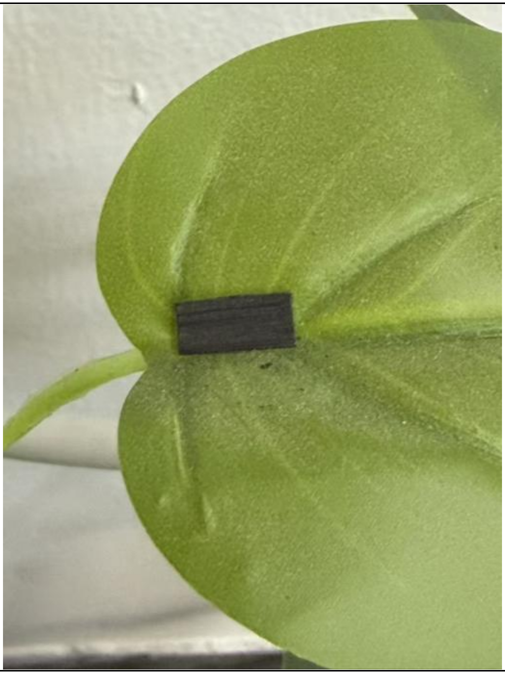

**Figure S1. Place the bamboo strips on the leaves.**

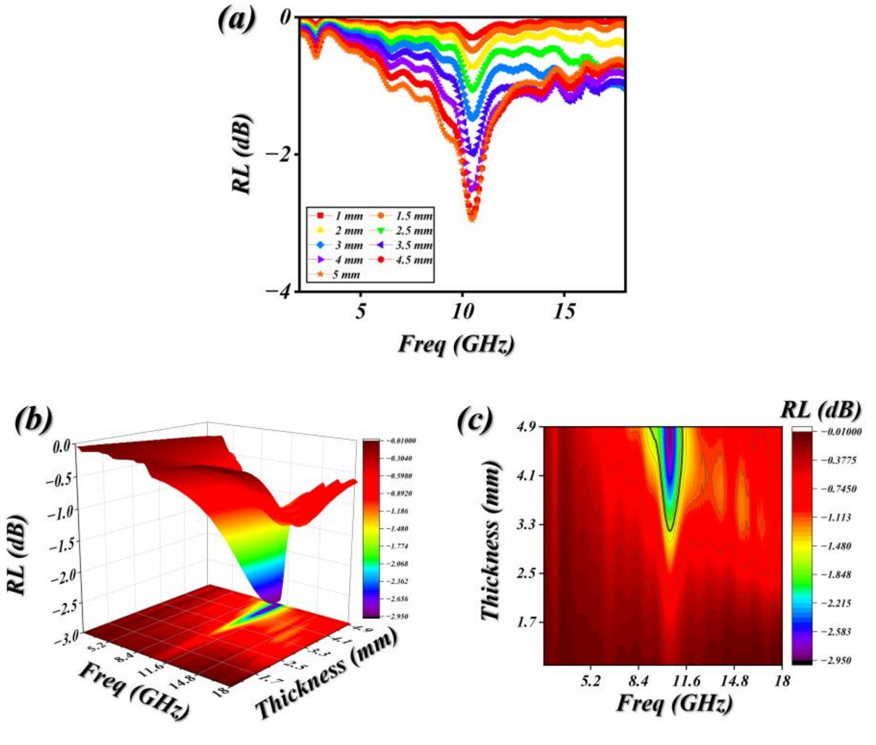


**Figure S2.** 2D and 3D RL results of BPC

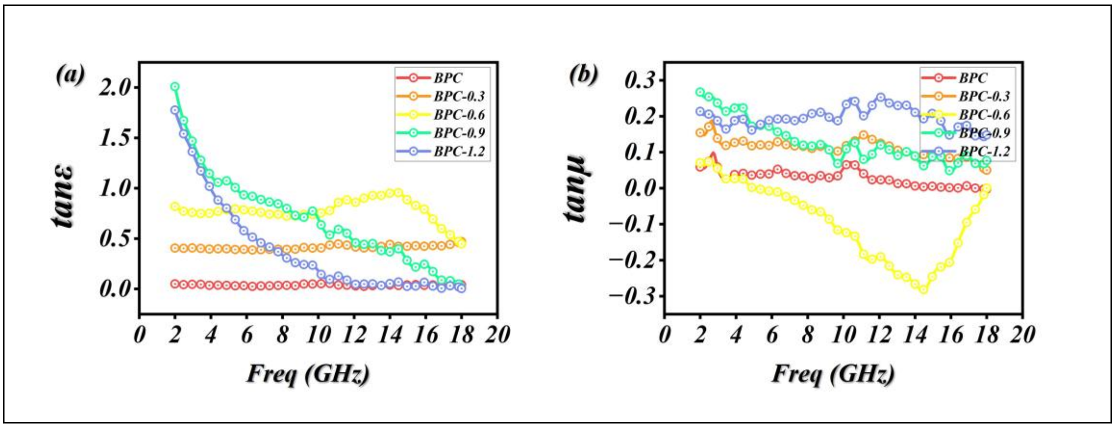


**Figure S3.** (a) Dielectric loss tangent angle and (b) magnetic loss tangent angle of BPCs

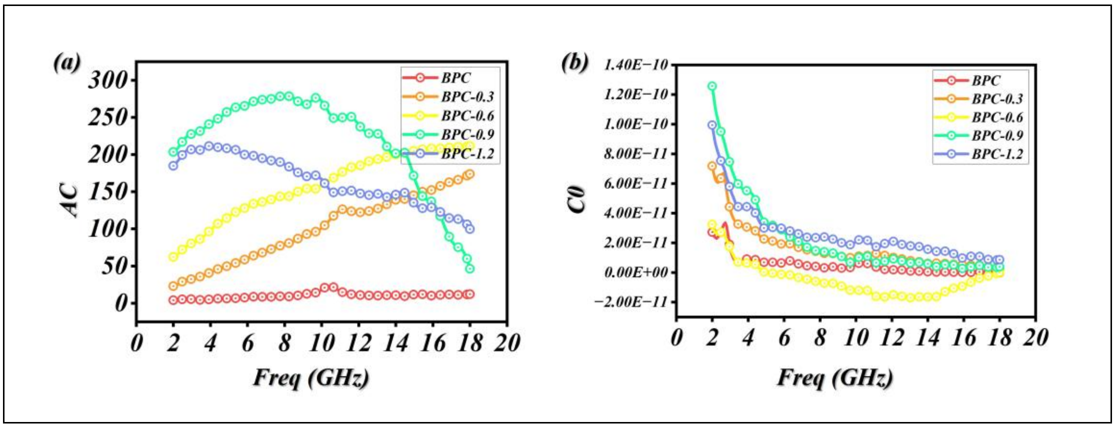


**Figure S4.** AC and C0 results of BPCs

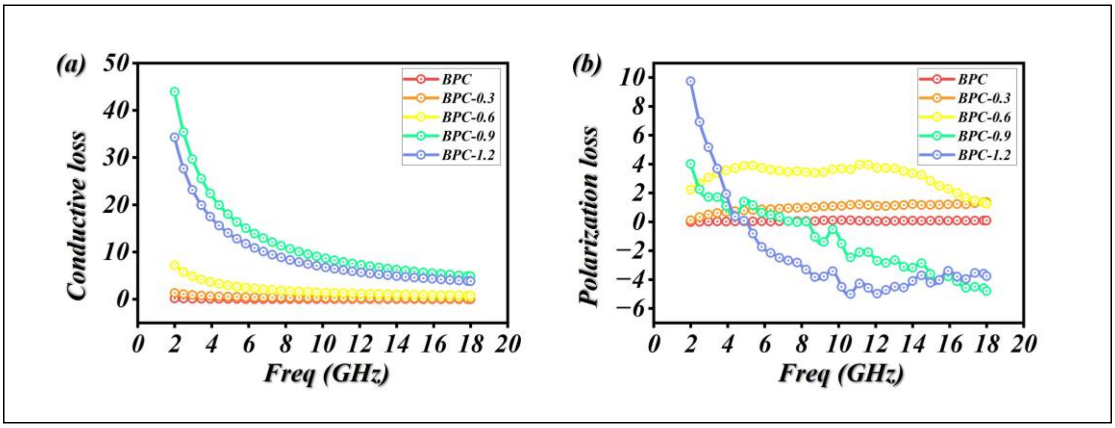


**Figure S5.** Conductive loss and polarization loss of different BPC specimens

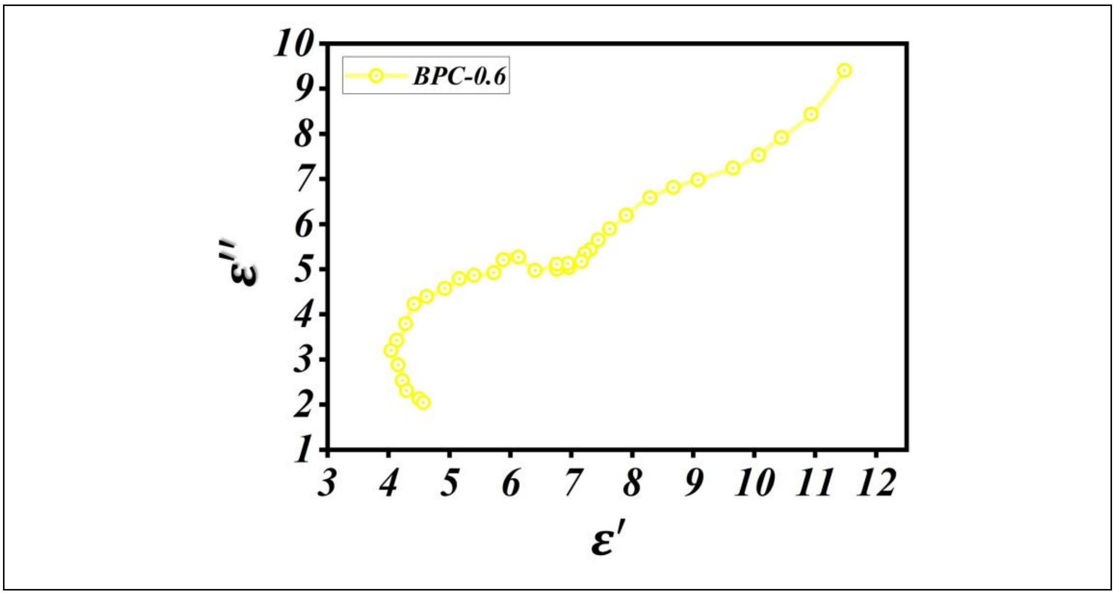

**Figure S6.** Cole-Cole curves of BPC-0.6

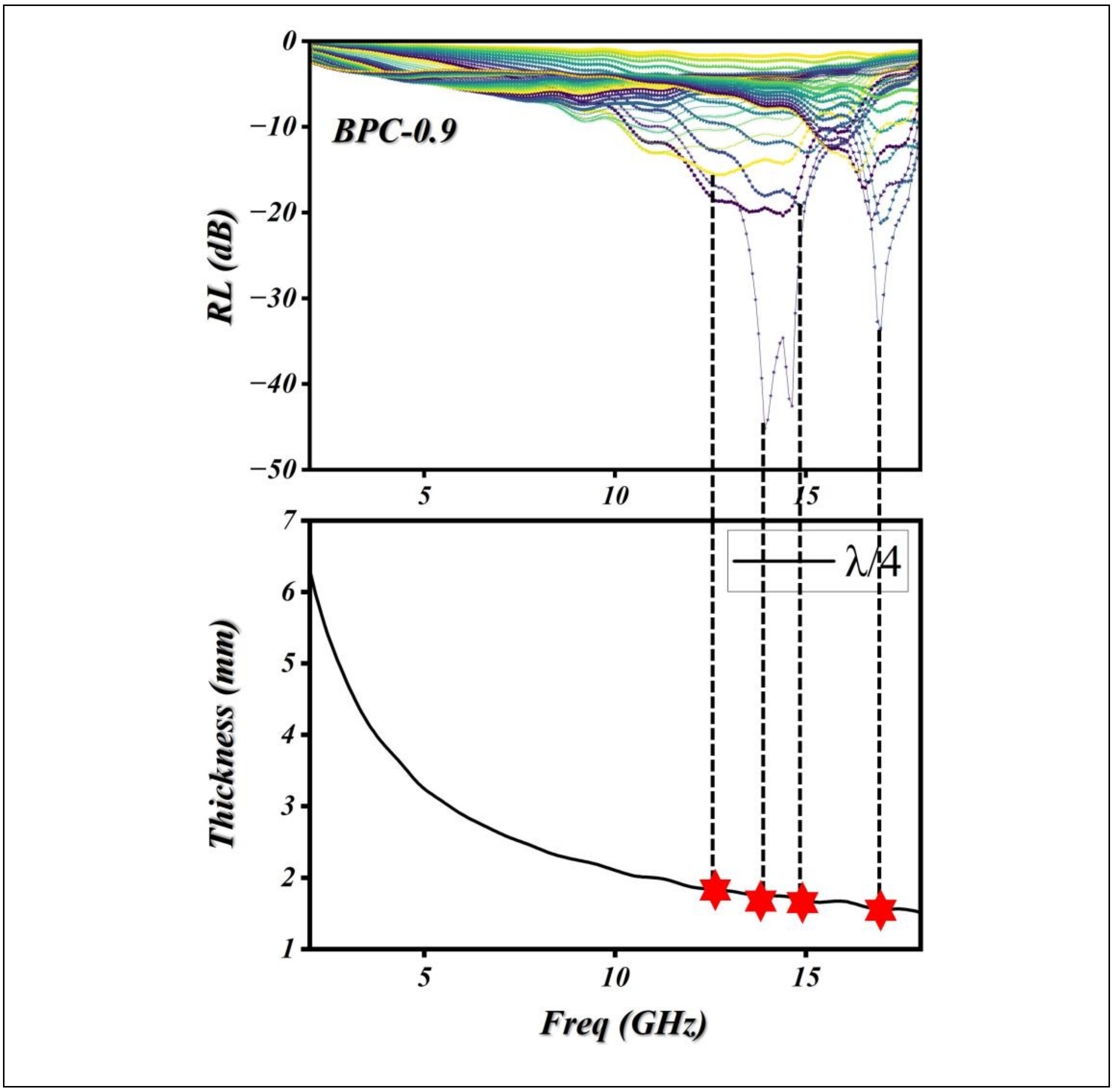

**Figure S7.** One-quarter wavelength model of BPC-0.9

**Table S1 Recent progress on biomass derived caron materials for microwave absorption, with a comparison to this work**

| Carbon type | Precursor | Tc (℃) | Frequency range (GHz) | EAB (GHz) | Dm(mm) | Absorption peak (dB) | Ref. |
|---|---|---|---|---|---|---|---|
| Traditional carbon | CNT | - | 2-18 | 4.4 | 2.00 | -47.6 | [1] |
| | RGO | - | 2-18 | 7.2 | 2.4 | -49.2 | [2] |
| | Graphene foam | - | | 4.56 | 3.5 | -53.9 | [3] |
| Biomass carbon | Fir wood | 690 | 2-18 | 7.63 | 3.73 | -16.3 | [4] |
| | Cotton | 800 | 2-18 | 14.2 | 2.00 | -43 | [5] |
| | Rice husk | 900 | 2-18 | 3.68 | 1.50 | -43 | [6] |
| | Walnut | 550 | 2-18 | 3.84 | 1.50 | -31.8 | [7] |
| | Bamboo | 1000 | 2-18 | 5.52 | 1.80 | -47.06 | [8] |
| | Wood | 1000 | 2-18 | 4.96 | 1.55 | -52.65 | [9] |
| | Soybean | 900 | 2-18 | 4.8 | 3.5 | -18.5 | [10] |
| | Waste Tire Rubbers | - | 2-18 | 4.2 | 3.5 | -47.43 | [11] |
| This work | Bamboo | 1000 | 2-18 | 6.65 | 1.8 mm | -45.17 | This work |

## Reference

[1] H. Sun, R. Che, X. You, Y. Jiang, Z. Yang, J. Deng, L. Qiu, H. Peng, Cross-Stacking Aligned Carbon-Nanotube Films to Tune Microwave Absorption Frequencies and Increase Absorption Intensities, Adv. Mater. 26 (2014) 8120–8125. https://doi.org/10.1002/adma.201403735.

[2] M. Zhang, X. Fang, Y. Zhang, J. Guo, C. Gong, D. Estevez, F. Qin, J. Zhang, Ultralight reduced graphene oxide aerogels prepared by cation-assisted strategy for excellent electromagnetic wave absorption, Nanotechnology 31 (2020) 275707. https://doi.org/10.1088/1361-6528/ab851d.

[3] P. Liu, Y. Zhang, J. Yan, Y. Huang, L. Xia, Z. Guang, Synthesis of lightweight N-doped graphene foams with open reticular structure for high-efficiency electromagnetic wave absorption, Chem. Eng. J. 368 (2019) 285–298. https://doi.org/10.1016/j.cej.2019.02.193.

[4] J. Xi, E. Zhou, Y. Liu, W. Gao, J. Ying, Z. Chen, C. Gao, Wood-based straightway channel structure for high performance microwave absorption, Carbon 124 (2017) 492–498. https://doi.org/10.1016/j.carbon.2017.07.088.

[5] W.-L. Song, Z. Zhou, L.-C. Wang, X.-D. Cheng, M. Chen, R. He, H. Chen, Y. Yang, D. Fang, Constructing Repairable Meta-Structures of Ultra-Broad-Band Electromagnetic Absorption from Three-Dimensional Printed Patterned Shells, ACS Appl. Mater. Interfaces 9 (2017) 43179–43187. https://doi.org/10.1021/acsami.7b15367.

[6] Q. Li, J. Zhu, S. Wang, F. Huang, Q. Liu, X. Kong, Microwave absorption on a bare biomass derived holey silica-hybridized carbon absorbent, Carbon 161 (2020) 639–646. https://doi.org/10.1016/j.carbon.2020.01.087.

[7] X. Qiu, L. Wang, H. Zhu, Y. Guan, Q. Zhang, Lightweight and efficient microwave absorbing materials based on walnut shell-derived nano-porous carbon, Nanoscale 9 (2017) 7408–7418. https://doi.org/10.1039/c7nr02628e.

[8] X. Huang, Y. Wang, Z. Lou, Y. Chen, Y. Li, H. Lv, Porous, magnetic carbon derived from bamboo for microwave absorption, Carbon (2023) 118005. https://doi.org/10.1016/j.carbon.2023.118005.

[9] C. Jia, T. Xia, Y. Ma, N. He, Z. Yu, Z. Lou, Y. Li, Fe3O4/α-Fe decorated porous carbon-based composites with adjustable electromagnetic wave absorption: Impedance matching and loading rate, Journal of Alloys and Compounds (2020) 157706. https://doi.org/10.1016/j.jallcom.2020.157706.

[10] J. Yue, J. Yu, S. Jiang, Y. Chen, Biomass carbon materials with porous array structures

derived from soybean dregs for effective electromagnetic wave absorption, Diamond and Related Materials 126 (2022) 109054. https://doi.org/10.1016/j.diamond.2022.109054.

[11] J. Zheng, M. Hanshe, W. He, T. Hang, Z. Li, S. Jiang, S. E, X. Li, Y. Chen, Highly Stretchable Composite Foams via Sustainable Utilization of Waste Tire Rubbers for Temperature-Dependent Electromagnetic Wave Absorption, Molecules 27 (2022) 8971. https://doi.org/10.3390/molecules27248971.
